# Effects of the Incorporation of Tannin Extract from Quebracho Colorado Wood on Color Parameters, Lipid Oxidation, and Sensory Attributes of Beef Patties

**DOI:** 10.3390/foods9050667

**Published:** 2020-05-21

**Authors:** Ana Paula B. Fruet, Francine M. Giotto, Mozart A. Fonseca, José Laerte Nörnberg, Amilton S. De Mello

**Affiliations:** 1 Department of Agriculture, Nutrition, and Veterinary Sciences, University of Nevada, Reno, 1664 N. Virginia St. mail stop 202, Reno, NV 89557, USA; ap_burin@hotmail.com (A.P.B.F.); fgiotto@cabnr.unr.edu (F.M.G.); mfonseca@cabnr.unr.edu (M.A.F.); 2 Department of Science and Food Technology, Federal University of Santa Maria, Santa Maria, 1000 Roraima Av., Santa Maria, RS 97105900, Brazil; jlnornberg@gmail.com

**Keywords:** beef, condensed tannin, Quebracho, lipid oxidation, color stability, sensory attributes, shelf life

## Abstract

The tannin extract of Quebracho Colorado wood (*Schinopsis balansae* and *Schinopsis lorentzii*) is rich in proanthocyanidins with demonstrated powerful scavenging activity against free radicals. Currently, this extract is used in the wine industry to improve sensory attributes, stabilize color, and act as a redox buffer. In this study, we hypothesized that condensed tannins from Quebracho Colorado wood could be incorporated into beef patties as a natural antioxidant source to improve shelf life. Patties formulated with tannin extract (0, 0.5%, 1%, and 1.5%) were evaluated for instrumental color, lipid oxidation, and sensory attributes. Patties were displayed under refrigerated aerobic conditions (PVC film) for 6 days for color and lipid oxidation analysis. For sensory analysis, patties were frozen immediately after formulation. Control (0%) samples were redder than samples formulated with 1.5% tannin during the first 4 days of display. For b*, samples formulated with 1.5% tannin were predominantly yellower during display. After day 4, chroma values were higher in samples formulated with 1.5% tannin. The inclusion of tannin extract improved lipid stability, however, levels above 0.5% decreased tenderness, softness, juiciness, and overall desirability of patties.

## 1. Introduction

Negative effects caused by lipid oxidation lead to detrimental effects on the flavor and shelf life of ground beef. The peroxidation of polyunsaturated fatty acids (PUFA) generates radicals that are further converted or decomposed to short-chain aldehydes, ketones, epoxides, hydroxyl compounds, oligomers, and polymers, compromising mainly the color and flavor of meats and meat products [1]. In order to minimize those effects on quality attributes and shelf life, synthetic or natural antioxidants may be added into meat and meat products to prevent lipid peroxidation, reduce the development of off-flavors, and improve color stability [2]. The mode of action of antioxidants in meat matrices consists in removing or sequestering oxygen and metal catalysts, preventing the formation of free radicals, avoiding the propagation of reactive oxygen species, and promoting chelation with transition metals [2,3,4].

Although synthetic antioxidants such as butylated hydroxyanisole (BHA) and butylated hydroxytoluene (BHT) have been commonly used in the formulation of meat products during the last decades, new trends associated with the consumer’s mindset towards health consciousness have increased the demand for natural ingredients, including antioxidants obtained from plant extracts [5,6,7]. Fruet et al. [8] demonstrated that beef patties formulated with natural antioxidant extracts obtained from herbs and fruits such as rosemary, acerola, lemon, lime, and orange showed better color and lipid stability when compared to patties formulated with no antioxidants. Natural extracts obtained from plants are rich in compounds with antioxidant properties, such as phenolic diterpenes, flavonoids, carotenoids and vitamin C [9,10,11], making them well suited for use in meat products as an alternative to replace synthetic antioxidants [6]. 

Recent research using a commercial hydrolysable tannin mixture (T 0125, Sigma Aldrich, St. Louis, MO, USA) suggested that tannins may be also used as a source of antioxidant for a variety of meat products including poultry, fish, and beef [12,13,14]. Tannins are water-soluble compounds that precipitate alkaloids, collagen, and other proteins and are usually classified as hydrolysable or condensed. Hydrolysable tannins contain alcohol and hydroxyl groups esterified by gallic or hexahydroxy diphenic acid, whereas condensed tannins are usually derived from flavan-3-ols and/or flavan 3,4-diols [15,16]. 

Condensed tannins obtained from Quebracho Colorado wood (*Schinopsis balansae* and *Schinopsis lorentzii*) are rich in astringent proanthocyanidins with demonstrated powerful scavenging activity against free radicals such as 2,2 diphenyl-1-picrylhydrazyl (DPPH) [17]. Quebracho-derived tannins are commonly obtained from the wood by using low-cost processes, including hot-water extraction and spray-drying, to generate a concentrated powder. The final powder is used in the tanning, cosmetic, pharmaceutical, and animal feed industries [18]. Currently, commercial extracts of condensed tannins from Quebracho wood have also been employed in the oenological industry to improve body and color and increase the bitterness of red wine, by reducing off-flavor caused by sulfur compounds. Those tannins also decrease oxidation and stabilize color through tannin–anthocyanin bounds. To our knowledge, there is no research reporting the effects of Quebracho-derived tannin as a novel ingredient to improve the quality of beef products. As previously mentioned, only a few studies have evaluated the effects of commercial mixtures of hydrolysable tannins derived from oak gall nuts on meat quality attributes. For this study, we hypothesized that tannins obtained from Quebracho Colorado wood could be used as a low-cost ingredient to be incorporated as an antioxidant in ground beef. In order to test our hypothesis, we evaluated the antioxidant effects of four levels of condensed tannins from Quebracho wood on instrumental color, lipid peroxidation, and sensory attributes to determine optimal levels of inclusion in ground beef.

## 2. Materials and Methods 

### 2.1. Sample Preparation

Beef trimmings (85%/15% lean fat ratio) were commercially acquired from Wolf Pack Meats, the University of Nevada, Reno, USDA-inspected harvest and processing facility, vacuum-packaged, and transferred under refrigerated conditions to the University of Nevada, Reno, Meat Quality Laboratory. After 5 days of aging, trimmings were ground using a 10-mm plate and divided into 4 large batches of 2 kg. Ground batches were formulated with 0.5%, 1%, and 1.5% of a commercial tannin extract (TE) obtained from Quebracho wood (*Schinopsis balansae* and *Schinopsis lorentzii*). A control level (0%) without the inclusion of tannins was also included as a treatment level. Each level of TE was incorporated into the ground beef batches and mechanically hand-mixed for approximately 2 min. The TE powder used in this study had a reddish-brown appearance, and its composition and instrumental color parameters are shown in Table 1. From each batch, forty patties weighing 120 g and approximately 1.5 cm in thickness were formed for color (n = 20, n = 5 for each treatment) and sensory analysis (n = 20, n = 5 for each treatment). For lipid oxidation, 20 aliquots of 30 g directly obtained from each treated batch were evaluated on day 1 (n = 5 for each treatment), whereas for day 4 (n = 20) and day 7 (n = 20) smaller patties (30 g each, 1.5 cm of thickness, n = 5 for each treatment) were used. A total of forty (n = 40) 120 g patties, forty (n = 40) 30 g patties and twenty (n = 20) 30 g ground beef aliquots were used in this experiment, which was replicated once. Samples assigned to be tested for lipid stability were immediately vacuum packaged and frozen at −80 °C after day 1, 4, and 7 until analysis could be done. 

### 2.2. Retail Display 

Samples analyzed for color stability and lipid oxidation were displayed in polystyrene trays overwrapped with an oxygen-permeable PVC film. The retail display was simulated in a glass door refrigerator (BioCold^®^) set at 4 ± 1 °C, equipped with fluorescent lights set at approximately 540 lux. During all 7 days, samples were randomly re-allocated daily inside the case to be uniformly exposed to uncontrolled variations that may occur in commercial refrigerated cases.

### 2.3. Color Analysis

Instrumental color was measured on day 1, 1 h after patties were fabricated (day 1), and subsequently daily during the next 6 days of display (7 days total). Color readings were obtained using a Minolta chromameter (CR-400, Konica Minolta Inc., Tokyo, Japan) and expressed as CIE Lab lightness (L*), redness (a*), yellowness (b*). In addition, Munsell parameters including hue angle and chroma value were also calculated:

Chroma = (a*^2^ + b*^2^)^1/2^ (1)

Hue = arctan (b*/a*)(2)

These parameters are associated with visual discoloration of beef since large hue angles are directly related to meat browning [19] and lower chroma values are associated with less color intensity and discoloration [20]. The sample surface was analyzed on three random locations of the patty and readings were averaged.

### 2.4. Lipid Stability

Lipid stability of ground beef patties was evaluated on days 1, 4, and 7 by following the thiobarbituric acid assay (TBA) described by Buege and Aust [21], modified by Ahn, Olson, Chen, Wu, and Lee [22]. Briefly, samples were pulverized using liquid nitrogen and stored at −80 °C. Subsequently, 5 g of pulverized sample was mixed with 14 mL of demineralized/deionized water and 1 mL of butylated hydroxianisole (BHA), homogenized and centrifuged at 200 rpm for 5 min. One ml of the homogenate was then transferred to a conical tube, mixed with 2-thiobarbituric acid/trichloroacetic acid (TBA/TCA), kept at 70 °C for 30 min, and centrifuged at 2000 rpm for 15 min. Aliquots of 200 μL were transferred to a well plate and absorbance was read at 540 nm (Synergy HT, Biotek, Winooski, VT, USA). Lipid oxidation (TBA values) was expressed as malonaldehyde (MDA) concentration (mg MDA/kg), and quantification was determined by comparing samples with standard concentrations of TEP (1,1,3,3,-tetra-ethoxypropane) diluted in glacial acetic acid ranging from 0 to 50 nmol/mL.

### 2.5. Sensory Analysis

Panel procedures were approved by the University of Nevada, Reno Institutional Review Board (IRB# 823708-1). Patties were thawed for 24 h at 4 °C and cooked on an electric grill (Hamilton Beach, Glen Allen, VA, USA). After the internal temperature reached 35 °C, patties were flipped and cooked until temperature reached 71 °C. Thermocouples attached to a temperature recorder datalogger (RDXL 12SD, Omega, Norwalk, CT, USA) were used to properly monitor the temperature. Cooked patties were cut into eight pie-shaped pieces and served warm to a 7-member sensory panel [23]. Four female and three female panelists with ages varying from 21 to 64 were trained according to Meilgaard, Civille, and Carr [24]. Panelists evaluated sensory attributes by using a 10-point scale for tenderness (0 = extremely tough and 10 = extremely tender), softness (0 = extremely chewy and 10 = extremely soft), juiciness (0 = extremely dry and 10 = extremely juicy), off-flavor intensity (0 = extremely mild and 10 = extremely intense), and overall desirability (0 = extremely undesirable and 10 = extremely desirable).

### 2.6. Statistical Analysis

In this experiment, data were analyzed as a completely randomized design. For instrumental color, the dataset was arranged in a 4 × 7 factorial design, whereas the fixed effects included TE inclusion and day of retail display. The following model was used:

*Y_ijk_ = µ + α_i_ + β_j_ + (αβ)_ij_ + ε_ijk_* (3)

where *Y_ijkl_* was the object color parameter value, *µ* was the grand mean across the treatments included in the experiment, *α*_i_ was the effect of TE inclusion from the grand mean specific to the *i* levels (0-control, 0.5, 1, and 1.5%), *β_j_* was the effect of day of retail display from the grand mean specific to *j* levels (1, 2, 3, 4, 6 and 7), and *(αβ)_ij_* was the interaction between both effects. The experiment error was also added into the model. The term *ε_ijk_* is the effect of errors. For lipid stability, the data was arranged as a 4 × 3 factorial with the 4 levels of TE inclusion but only 3 levels of day of retail display (1, 4, and 7). Both color and lipid stability were analyzed as repeated measures considering the best covariance matrices indicated by the smallest Akaike and Bayesian information criteria (AIC and BIC, respectively). For sensory analysis, TE inclusion was considered the main effect and panelist a random effect. The following model was used:

*Y_ijk_ = µ + α_i_ + ε_ijk_* (4)

where *Y_ijk_* is the score for each sensory attribute, *µ* is the grand mean across the treatments included in the experiment, *α*_i_ is the effect of TE inclusion from the grand mean specific to the *i* levels (0-control, 0.5, 1, and 1.5%) and *ε_ijk_* is the effect of errors. Data were analyzed using the GLIMMIX procedure of SAS^®^ (9.4 package, SAS Institute, Inc., Cary, NC, USA), and when comparisons were significantly different at *p* ≤ 0.05, LSMEANS and DIFF functions were used to separate the means.

## 3. Results

### 3.1. Lipid Stability

**Figure 1:**
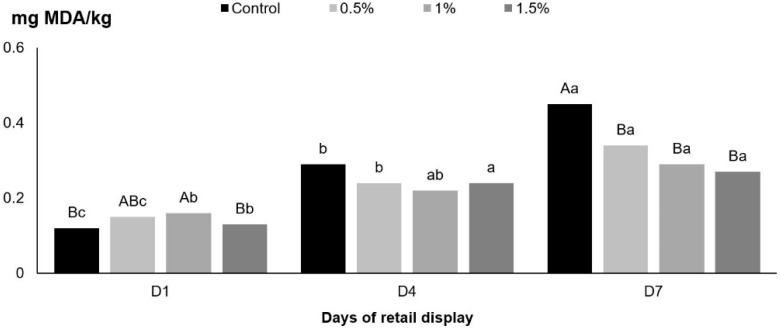
TBA values (mg MDA/kg) of beef patties formulated with different levels of TE (Control = 0%, 0.5%, 1%, and 1.5%). *p* = 0.002, Standard error of the mean = 0.01. ^A,B^ Means with different superscripts are significantly different within days of retail display. ^a,b,c^ Means with different superscripts are significantly different within tannin extract level.

For lipid stability, there was an interaction between both fixed effects of days of retail display and TE inclusion (*p* = 0.002) (Figure 1). On day 1 of display, small differences were observed among the TE treatments. Patties treated with 1% TE had higher MDA values when compared to patties treated with 0% and 1.5% TE. On day 4 of display, TE inclusion did not affect lipid stability. However, on day 7, all samples formulated with TE showed better lipid stability when compared to samples formulated with no TE (0%).

When looking at individual treatments, patties treated with 1% and 1.5% TE extract had similar lipid stability on days 4 and 7 of display. For patties treated with 0% and 0.5%, lipid oxidation gradually increased during the display time. However, on day 7, all samples treated with TE had improved lipid stability when compared to control samples (0%).

### 3.2. Instrumental Color

The effects of TE inclusion and day of retail display on instrumental color parameters are shown in Table 2. Inclusion of TE did not alter the lightness of patties (*p* = 0.169). Values of L* decreased during the display time (*p* < 0.001). Interactions between both fixed effects were observed for a*, b*, and chroma (*p* < 0.001). The inclusion of TE affected a* values during the first 5 days of display, whereas after day 6, redness was similar on patties from treatments. During the first two days, as inclusion levels increased, redness decreased. On days 3 and 4, all patties formulated with any level of TE were less red than control patties (0%). On day 5, control patties and patties formulated with 1% TE were redder when compared to patties formulated with 1.5%. Overall, TE inclusion compromised redness during the first 5 days of display, but it did not affect this parameter on the last two days of retail. For b*, patties formulated with 1.5% TE were the yellowest from the second to the last day of retail display. During the whole display period, as levels of TE decreased, yellowness also decreased. Patties formulated with 1.5% TE were consistently yellow during the whole display time. Chroma values decreased during display time. Inclusion of TE led to lower chroma values on days 2 and 4 and high values on day 7. Samples formulated with 1.5% TE showed higher chroma values at the end of the display time (days 6 and 7) when compared to control samples (0%). Hue angle increased during display. Overall, samples formulated with TE showed larger angles when compared to control samples. As levels of TE increased, hue angle values gradually increased. Samples formulated with 1.5% showed the largest angle.

### 3.3. Sensory Attributes

**Figure 2:**
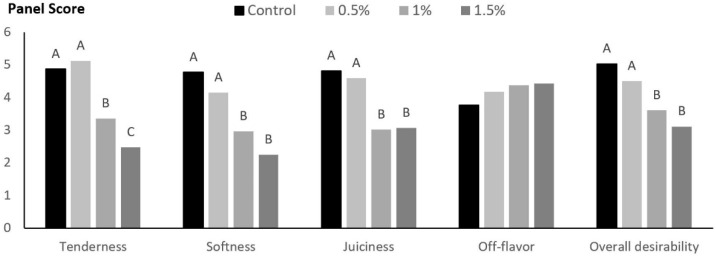
Sensory attributes of beef patties formulated with different levels of TE (Control = 0%, 0.5%, 1%, and 1.5%). Tenderness *p* < 0.0001, SEM = 0.10; softness *p* < 0.0001, SEM = 0.11; juiciness *p* < 0.0001, SEM = 0.12; off-flavor *p* = 0.353, SEM = 0.13; and overall desirability *p* < 0.0001, SEM = 0.11. ^A,B,C^ Means with different superscripts are significantly different within tannin extract levels.

Except for off-flavor intensity, levels of TE above 0.5% led to significant effects on all sensory attributes, (Figure 2). Trained panelists scored samples formulated with both TE concentrations (1% and 1.5%) as less tender, chewier, less juicy, and less desirable, whereas samples formulated with 1.5% received the lowest tenderness scores. Samples formulated with no TE (control) and 0.5% received similar scores for all attributes.

## 4. Discussion

Tannins are generally recognized as safe (GRAS) and can be used as a food additive when formulating processed meat products [25]. Previous research showed that ground beef formulated with hydrolysable tannins diluted in water (200 mg/kg) [14] had improved lipid stability. In this study, using a different type of tannin (condensed), similar results were observed regarding lipid stability. On day 7, all samples formulated with TE showed lower lipid oxidation when compared to control samples. Condensed tannins differ from hydrolysable tannins mainly in composition [26]. Condensed tannins, or proanthocyanidins, are composed by several derivatives of flavonoids (flavan 3-ol nuclei) classified according to hydroxylation patterns. Proanthocyanidins are usually divided into two groups, a larger group formed by procyanidins and a smaller group of prodelphinidins [27]. Hydrolysable tannins, on the other hand, are composed of a core of glucose and are usually referred as Pentagalloyl glucose (PGG). The core is usually linked to gallic acid (gallotannins) or to hexahydroxydiphenic acid (ellagitannins) [28]. Overall, antioxidant properties of all tannins are associated with mechanisms such as scavenging activity, chelation of transition metals, and inhibition of prooxidative enzymes. Previous research showed that procyanidins (condensed tannins) have stronger antioxidant properties than ascorbic acid and α-tocopherol [29]. Reports using natural ingredients that contain tannins, such as peanut byproducts and sorghum flour, demonstrated that tannins can be incorporated into meats without causing detrimental effects on quality attributes [30,31]. The inclusion of an extract of peanut butter in ground beef also improved lipid stability [32]. In addition, condensed tannins from quebracho wood were reported to be effective nitrogen oxide scavengers [18], suggesting that proanthocyanidins can be successfully used as efficient antioxidants. Although research suggested that PGG (hydrolysable tannin) is also an efficient antioxidant [14], there is no research that directly compares the antioxidant effects of condensed versus hydrolysable tannins incorporated in food matrices. In this study, the application of condensed tannins in ground beef formulation successfully demonstrated that this type of tannin can also be used as a functional food ingredient to improve lipid stability.

The inclusion of any level of Quebracho-derived tannins did not affect lightness (L*) of beef patties. However, interactions between treatment and day were observed for a*, b*, and chroma values. Loss of redness (a*) and further discoloration are usually associated with hemoglobin-mediated lipid oxidation in muscle [33]. It is well known that redness is directly correlated to the presence of oxymyoglobin in meats. In the oxymyoglobin state, the heme group of myoglobin containing the ferrous iron binds to oxygen. This pigment is responsible for the bright cherry-red color commonly observed in fresh beef. When oxidized, the iron of the heme group of myoglobin becomes ferric, binding to a hydroxyl radical forming metmyoglobin, a pigment associated with meat browning and discoloration [20]. Previous research demonstrated that byproducts of lipid oxidation, including α and β unsaturated aldehydes and 4-hydroxynonenal, especially from peroxidation of polyunsaturated fatty acids, detrimentally affect color by promoting the formation of metmyoglobin [19,34]. Therefore, as lipid oxidation occurs, it is expected to produce lower values of a*, lower chroma values and large hue angles. The Munsell visual parameters chroma and hue angle express nuance and color saturation [35]. Lower values of chroma indicate less color intensity and a large hue angle indicates meat browning [36,37]. In this study, although we observed lower lipid oxidation in patties formulated with TE from days 2 to 4 of retail display, inclusion of TE led to lower redness. Similarly, chroma values were also lower during the same period, but higher on day 7. Overall, as inclusion levels of tannic acid increased, larger hue angles were observed. Therefore, instrumental color parameters presented in this study seem to not be aligned with lipid oxidation results, which were lower in patties formulated with TE. These negative correlations are possibly associated with the instrumental color parameters of the powder (Table 1). The TE tested in this study had a reddish-brown appearance with low a*, b* and chroma, and high L* and hue angle values. Since lower lipid oxidation was similar in all patties on day 4 and lower in patties formulated with tannins on day 7, instrumental color results suggest that the color of the extract may have contributed to lower a* and chroma and higher hue angle values in treated patties. Although in this research authors did not objectively quantified metmyoglobin concentration, previous research reported that when ground beef was packed in a high-oxygen modified atmosphere, samples treated with tannic acid had higher oxymyoglobin concentrations when compared to untreated samples [14]. In addition, inclusion of tannic acid in poultry also reduced overall protein oxidation [12]. This suggests that the effects of the inclusion of Quebracho-derived tannins on color may be exclusively associated with its color characteristics and not with any factor that induces myoglobin oxidation. Overall, values of yellowness increased as levels of TE increased. In beef, values of b* are usually smaller when compared to L* and a* and, possibly, less susceptible to variations caused by the color of the TE powder.

Regarding sensory attributes, although no significant differences in off-flavor intensity were detected by panelists, patties formulated with levels above 0.5% TE were significantly tougher, chewier and less juicy. Overall, patties formulated with 1% and 1.5% TE received lower desirability scores. In this study, TE was used directly as a powder and not diluted in water as previously reported in other research [12,13,14]. In the United States, retained water present in ground beef must be declared on the label [38]. Therefore, by using the powder, our goal was to avoid the need for declaring any possible retained water on the final product label. However, including levels above 0.5% directly affected some of those sensory attributes. The powder state and flavor of the TE used in this study possibly contributed to the lower scores of overall desirability and juiciness when TE levels increased. Although panelists did not report differences in off-flavor intensity, proanthocyanidins are flavanol polymers with astringent, bitter, and sour properties [39], which may have compromised the overall desirability of patties formulated with levels above 0.5%. Conversely, it was expected that TE inclusion would improve juiciness perception since astringency is a potent saliva flow elicitor [40]. However, the addition of dry ingredients may directly affect tenderness, softness, and juiciness. Previous research reported that patties formulated with breadcrumbs and soy protein isolate were found to be chewier and less tender when compared to patties formulated with beef only [41,42]. The TE powder used in this research had 82.5% dry matter, and was thus a very dry ingredient.

## 5. Conclusions

The inclusion of condensed tannins obtained from Quebracho Colorado wood improved lipid stability in ground beef. However, when added as powder, detrimental effects on product color and sensory perception may occur. Levels of inclusion above 0.5% negatively impacted quality attributes. Visual differences may be noticed if the product is displayed fresh packaged in permeable films. Additionally, effects on sensory attributes may lead to less desirability. If used as the only antioxidant source, the inclusion of Quebracho-derived tannins as antioxidants in ground beef must be performed with caution to avoid exceeding optimal levels. However, further studies are needed to compare condensed tannins against other natural antioxidants and to approach possible synergistic interactions with other antioxidant sources. Due to their low cost, condensed tannins could be combined with other natural extracts such rosemary, acerola and other natural sources to decrease ingredient costs.

## Figures and Tables

**Table 1:** Tannin extract (TE) composition (%) and instrumental color.

	Dry Matter	Moisture	pH ^2^	Ashes	L*	a*	b*	Chroma	Hue Angle
TE ^1^	82.5	8.0	4–5	4	40.3	9.8	9.06	13.35	42.75

^1^ Profisetinidin condensed tannin obtained from Quebracho Colorado wood (*Schinopsis balansae* and *Schinopsis lorentzii*). ^2^ Water solution 10%.

**Table 2:** Effect of tannin extract (TE) inclusion and days at display on color parameters of beef patties (avg = average of TE level or day of retail display).

		Days at Display-*β*							
	TE-*α*	1	2	3	4	5	6	7	avg
L*	0%	51.07	47.70	49.17	48.25	48.12	50.38	45.90	48.65
	0.5%	48.24	46.04	47.86	48.51	47.37	48.29	45.45	47.39
	1%	48.85	47.38	48.52	46.99	48.11	47.41	46.29	47.65
	1.5%	50.33	49.28	49.24	48.23	48.88	48.82	47.78	48.94
	**avg**	49.62 ^a^	47.60 ^cd^	48.70 ^b^	47.99 ^bc^	48.12 ^bc^	48.72 ^b^	46.36 ^d^	
a*	0%	20.56 ^Aa^	18.60 ^Ab^	17.24 ^Ac^	16.92 ^Ac^	14.34 ^Ad^	12.21 ^e^	12.37 ^e^	16.03
	0.5%	19.83 ^ABa^	16.79 ^Bb^	15.57 ^Bc^	14.35 ^Bd^	12.96 ^Be^	12.06 ^e^	12.59 ^e^	14.86
	1%	19.72 ^ABa^	15.51 ^Cb^	15.40 ^Bb^	14.38 ^Bc^	13.28 ^ABc^	12.16 ^d^	13.02 ^cd^	14.78
	1.5%	19.40 ^Ba^	15.91 ^BCb^	15.42 ^Bb^	14.10 ^Bc^	13.20 ^Bcd^	12.38 ^d^	12.71 ^d^	14.73
	**avg**	19.89	16.70	15.91	14.94	13.45	12.20	12.65	
b*	0%	12.88 ^BCa^	11.61 ^Bb^	11.85 ^Bb^	11.83 ^Bb^	10.67 ^Cc^	10.53 ^Bc^	9.80 ^Cc^	11.31
	0.5%	12.22 ^Ca^	10.47 ^Cbc^	11.39 ^Bab^	11.33 ^Bab^	10.75 ^Cbc^	10.67 ^Bbc^	10.18 ^BCc^	11.00
	1%	13.34 ^ABa^	10.95 ^BCd^	12.23 ^Bb^	11.15 ^Bcd^	12.02 ^Bbc^	11.37 ^Bbcd^	11.25 ^Bbcd^	11.76
	1.5%	14.08 ^Aa^	12.83 ^Ab^	13.82 ^Aab^	12.97 ^Ab^	13.73 ^Aab^	13.19 ^Aab^	13.16 ^Aab^	13.40
	**avg**	13.13	11.46	12.32	11.81	11.79	11.44	11.10	
**Chroma**	0%	24.27 ^a^	21.93 ^Ab^	20.92 ^Abc^	20.65 ^Ac^	17.90 ^ABd^	16.14 ^Be^	15.80 ^Ce^	19.66
	0.5%	23.31 ^a^	19.78 ^BCb^	19.29 ^Bbc^	18.29 ^Bc^	16.86 ^Bd^	16.11 ^Bd^	16.18 ^BCd^	18.54
	1%	23.82 ^a^	19.00 ^Cbc^	19.67 ^ABb^	18.20 ^Bcd^	17.93 ^ABcd^	16.66 ^Be^	17.21 ^ABde^	18.93
	1.5%	23.00 ^a^	20.44 ^Bb^	20.72 ^Ab^	19.16 ^Bc^	19.05 ^Ac^	18.10 ^Ac^	18.31 ^Ac^	19.97
	**avg**	23.85	20.29	20.15	19.07	17.94	16.75	16.88	
**Hue**	0%	32.08	31.97	34.45	34.95	36.72	40.87	38.41	35.64 ^D^
	0.5%	31.62	31.94	36.17	38.31	39.81	41.54	39.35	36.96 ^C^
	1%	33.99	35.17	38.47	37.7	42.13	43.03	40.81	38.76 ^B^
	1.5%	35.92	38.87	41.81	42.57	46.12	46.83	46.01	42.59 ^A^
	**avg**	33.40 ^e^	34.49 ^d^	37.73 ^c^	38.38 ^c^	41.19 ^b^	43.07 ^a^	41.14 ^b^	

^ABC^ Means with different superscripts are significantly different within TE levels (*α*). ^abcde^ Means with different superscripts are significantly different within days of retail display (*β*). For L*: Standard error of the mean (STE) = 0.113, *p* values *α* = 0.169, *β* < 0.0001, and *αβ* = 0.075. For a*: STE = 0.061, *p* values *α* < 0.0001, *β* < 0.0001, and *αβ* = 0.004. For b*: STE = 0.055, *p* values *α* < 0.0001, *β* < 0.0001, and *αβ* = 0.047. For chroma: STE = 0.067, *p* values *α* < 0.0001, *β* < 0.0001, and *αβ* = 0.001. For Hue: STE = 0.152, *p* values *α* < 0.0001, *β* <0.0001, and *αβ* = 0.288. For hue angle, no interaction was detected, and means identification was performed for avg of single effects (*p* < 0.0001).
